# Additional Observations on Amputation

**Published:** 1788

**Authors:** James Lucas

**Affiliations:** one of the Surgeons of the General Infirmary at Leeds, and Member of the Corporation of Surgeons in London


### THE LONDON MEDICAL JOURNAL.

I. *Additional Obſervations on Amputation. Communicated in a Letter to Dr.* Simmons, *F. R. S. by Mr.* James Lucas, *one of the Surgeons of the General Infirmary at Leeds, and Member of the Corporation of Surgeons in London.*

FROM a reconſideration of thoſe obſervations on amputation * you have done me the honour to communicate to the Public through the channel of the London Medical Journal; and the objections that have been made by others to ſome parts of the practice; as well as from the additional experience I have ſince had, I am induced to requeſt an inſertion of ſome farther remarks on the ſubject.

It has been obſerved, that the flap operation does not ſucceed, and that the ſecondary union of the flap is preferable to placing it in immediate contact. Some have objected to Mr. Alanſon's plan, from the difficulty of making the

* See Vol. VII. page 225, and Vol. VIII. page 142.

ſloping floping incifion through the mufcles; whilft others have thought this difficulty a fufficient reafon for totally difcontinuing his improvements. His after-treatment has alfo been cenfured for procuring the union too haftily, and fubjecting the healing of the wound to be impeded by the formation of matter, as well as for producing a difficulty in the cafting off of the ligatures.

Having been a practitioner of this operation at an hofpital for twenty years, and having made trial of every method that appeared to the furgeons, in confultation, an improvement, as well as having paid peculiar attention to this fubject, I feel myfelf able to write upon it with fome precifion; and hope to elucidate what I have already advanced by enumerating fuch cafes as appear to me moft explanatory of the practice I have ventured to recommend.

Of not lefs than fixty or feventy amputations, only four or five have been done immediately after the accident; nor have the deaths exceeded that number, and thefe have been all in chronic cafes; not one has happened fince we united ftumps by the firft intention. It, therefore, cannot be faid that we have been advocates for fpeedy dy amputation, or that our practice has been unſucceſsful.

On looking over my notes concerning the operations that happened at the hoſpital I attended in London, I find, that, of nine patients who underwent amputation chiefly for chronic diſeaſes, four died. As the ſurgeons were of great eminence in their profeſſion, much of this want of ſucceſs muſt be attributed to the bad air of a crowded hoſpital in the center of a populous city; and, in the cauſes of this fatality, the intemperance of ſome of the patients might alſo have a ſhare.

The intention of my former paper was neither to recommend precipitate amputation; nor that the expreſſion * reſpecting the life of the patient being *immediately* concerned ſhould have any relation to chronic caſes. It was not written merely to revive any particular operation, but to give a ſketch of ſuch practice as appeared to me to have merit; as well as to point out ſuch deviations from it as had come to my knowledge, and had been probable cauſes of failure.

I confeſs myſelf ſtill an advocate for the flap operation, in certain caſes, in preference to the

* Vol. VII. page 225.

eſtabliſhed eſtabliſhed circular inciſion. In amputations near the ancle, the flap, united by the firſt intention, is, of all other operations, the moſt ſatisfactory. The facility with which it unites, its peculiar neatneſs, and future advantage, are ſtrong reaſons for preſerving, not only in this but in all other ſimilar operations, a due proportion of muſcular parts. From continuing to obſerve *conſtant and unremitted ſucceſs* attend the uniting the flap immediately; and from a perfect recollection of the additional pain at each dreſſing, as well as the delay occaſioned by the ſecondary union, I cannot, in too ſtrong terms, repeat the merit that is due to the ſpeedy cure.

Before, when we were in the habit of dreſſing the flap and ſtump ſeparately*, the patients begged "they might not be flapped," from the violent pain they underſtood others had felt; whereas, ſince the flap has been united immediately, ſeveral have particularly requeſted that the operation might be done with a flap.

In the ſtrong muſcles near the knee there is more difficulty in forming and adapting a flap;

* See Medical Obſervations and Inquiries, Vol. V. page 323, where I have given my reaſons for having formerly adviſed that practice.

but but a much better ſtump may in that part be procured by an oblique inciſion, reſerving a greater proportion of cellular and muſcular parts, ſo as nearly to meet the anterior teguments.

If the parts above the knee are ſound, we prefer Mr. Alanſon's operation; but if they are diſeaſed, or ulcerated, and yet there can be choſen a ſound flap, whether it be anterior, poſterior, or lateral, we find the amputation with a flap more eligible, and equally ſucceſsful.

The ſloping inciſion through the muſcles, directed by Mr. Alanſon, requires the operator to cut with that edge of the knife near its point, as I have already obſerved * in my former paper; where I have alſo added, that a defect in the execution of it, ſo far from being a ſufficient reaſon for diſcontinuing the plan, produces no material alteration either in the cure or future benefit; nor have I omitted † to ſpeak fully to the poſſible objections.

In all the methods of uniting wounds by the firſt intention, we prefer the ſaving ſo much of the ſoft parts, that the edges of the wound

* Vol. VII. page 230.

† Ibid. page 241.

may meet with great facility, and keeping them in contact by paſſing, with a round needle, as many ſutures as appear neceſſary to ſupport the union; and ſo confident are we of its being totally unneceſſary to leave any opening for the diſcharge of matter, (farther than is requiſite for the impending ligatures) that we unite the intervening ſpaces with ſhort ſlips of court plaſter.

In many inſtances there will be found ſome maturation at the firſt dreſſing; but this circumſtance ſhould not induce us to uſe means to encourage it, or to deſiſt from retaining the lips of the wound in contact, as the perfect adheſion of the parts will not be obſtructed.

The diſcharge will be more or leſs in proportion to the number of ligatures; to the quantity of ſubſtance included with the artery, or of blood either left on the ſtump, or oozing from it after it has been dreſſed: it may depend alſo on the cellular parts being more or leſs diſeaſed. But in no inſtance whatever have I ſeen any failure from theſe circumſtances; or any other benefit from leaving the edges of the wound farther ſeparated, (although that has occaſionally been tried) except this, that the ligatures, I have thought, were removed rather more eaſily, and

ſooner.

fooner. But this never feemed to compenfate for the additional pain fuffered by the patient at each dreffing of the ftump.

For fome time we united the edges of the wound with long and broad flips of adhefive plafter, fpread on leather, at the firft dreffing; but we found that the whole limb was rendered more uneafy, and the edges of the wound inflamed more, than fince we have adopted the method of ufing futures and fhort flips of court plafter. Still, however, when the futures are removed, we find a long flip of adhefive plafter, as broad as the flap, ufeful in fupporting a ftrict adhefion; but in Mr. Alanfon's method the continuance of the long tow pledgets is, in general, a fufficient fupport.

Since we have difcovered the dimenfions requifite *, and marked the parts for incifion, no difficulty has arifen in adapting them with exactnefs; and fince the apparatus has been difpofed according to an alphabetical lift marked upon a card, which is conftantly kept with the inftruments, no perplexity has happened from any article having been forgotten; and I ftill think thefe minutiæ by no means unworthy the notice

* Vol. VII. page 229.

of any practitioner, particularly of fuch as are not in the habit of performing operations.

I have not, for fome time, had occafion to ufe a retractor, which I have found liable to feparate the periofteum above the part where the bone is fawn through ; and I ftill prefer the faw being held with an elevated hand, rather than fawing through the bone horizontally.

### CASE I.

In November, 1765, whilft I was a dreffing pupil at St. Bartholomew's hofpital, in London, I was called to a compound fracture of the tibia and fibula, within four inches of their lower extremity. Not only the bones were violently broken, but the mufcles were much torn. Mr. Crane, who was foon after fent for, took no fmall pains to perfuade the man to lofe his limb, as the only chance of preferving his life. Being unable to prevail, he removed two inches of the tibia, placed the limb in an extended ftate, and gave fuch directions as he thought neceffary.

The next morning the patient informed me, that for feveral hours he had fuffered more than he could poffibly have done from an operation, to which he was therefore now defirous of fubmitting ; mitting; but Mr. Crane was of opinion that ſuch a ſtep could only tend to haſten his death, which happened on the third day after the accident.

### CASE II.

A Coalminer was admitted into the Leeds Infirmary with his leg terribly ſhattered by a fall of coals, ſeveral pieces of which had penetrated into and were intermixed with the muſcles. At firſt the bleeding was violent, but it gradually abated. Cooling remedies were applied, and a tourniquet was kept in readineſs. The hæmorrhage returned every or every other day, but always ceaſed before any artery, from whence it came, could be diſcovered. Although every attention was paid by a perſon placed to watch the limb, the patient died in ten days. Neither the habit nor ſtate of his limb were ſuch as to render amputation adviſeable, unleſs it had been done early.

A patient of mine, who was not brought into the Infirmary until ſome days after a compound fracture of the leg, ſunk under the diſcharge and fever before his limb became in a condition to warrant an operation; whilſt another man, admitted a few days after the accident, and apparently rently ſinking, underwent an unſucceſsful amputation.

### CASE III.

A Coalminer, whoſe leg was much ſhattered about the middle, by having two or three inches of it removed, after ſtruggling through many difficulties from abſceſſes, exfoliations, and great fever, gradually recovered, and had in the end an uſeful limb.—This man was confined to his bed near four months.

### CASE IV.

James Walker, upwards of ſixty years old, was admitted into the Infirmary with a very bad compound fracture, which prevented him from being able to have his bed made for near nine months. At firſt every attempt was made to unite the wound by the firſt intention; but a ſuppuration ſoon prevented ſuch an effect. He was repeatedly in imminent danger, and often expreſſed a wiſh ſtill to have his leg taken off. He did in time recover; but for ſome years his limb has continued of little uſe to him.

### CASE V.

A middle-aged man had the misfortune to have both bones of his leg, near the knee, broken ken by a loaded carriage. There was a wound communicating with the fracture; and from this aperture, which was not larger than to admit the blunt end of a probe, flowed a ſtream of blood, as when a vein in the arm is opened. The wound was dilated, with a view to diſcover from whence the blood came, or to remove any ſtricture that might occaſion it, but in vain. From the quantity of oil mixed with the blood, the medullary artery was ſuppoſed to be wounded. As the hæmorrhage could be almoſt entirely reſtrained, when the hand was preſſed upon a piece of ſponge applied to the wound, it was agreed that ſuch preſſure ſhould be made until the bleeding was ſtopped. It was more than forty-eight hours before that was accompliſhed; yet it ſucceeded, and the cure was very ſatisfactory. The violent ſwelling of the knee and parts adjacent would, in this caſe, have rendered an operation equally difficult, had we been unable to have checked the bleeding.

### CASE VI.

Eſther Pearſon, aged ſeventy-three years, was admitted into the Infirmary, as my patient, with a compound fracture of each leg, from a coal waggon paſſing over them. One of her limbs was was taken off above the knee immediately, according to Mr. Alanſon's method; and an account of the operation is given both by Mr. Hey and myſelf in the ſecond edition of Mr. Alanſon's work on amputation.

About four inches of the tibia of the other leg were removed, and due pains taken to make the poor woman as comfortable as her deplorable ſituation would allow. The limb was ſo ſtarved, that a warm cataplaſm became needful; otherwiſe I uſually apply ſoap cerate, or saturnine lotion at the firſt.

After a confinement in bed of upwards of ten months, various attempts were made to ſupport her upon crutches; but, after trying for a few weeks, ſhe endured ſo much pain, that ſhe begged for the removal of a limb that was to a degree burdenſome, without a proſpect of any amendment. On its being agreed to comply with her requeſt, a doubt was entertained whether the amputation ſhould be performed above or below the knee. The latter was unfortunately made choice of; for although an uncommon number of arteries were ſecured by ligatures, yet there ſtill continued a very menacing bleeding from the ſurface of the ſtump, until the edges of the wound were, by long ſlips of adheſive five plaster, drawn as nearly into contact as they would admit. Although this pressure appeared to be beneficial, yet in less than four hours the hæmorrhage returned with such violence, that I found the woman scarce able to speak. The dressings being immediately removed, not a single vessel requiring a ligature could be discovered, and yet the bleeding continued from the whole surface of the stump so violently as to render some instantaneous check necessary. Accordingly large pieces of sponge, sufficient to cover nearly the whole of the naked stump, were applied, and by the hand pressed with some force for upwards of twenty-four hours before the bleeding would otherwise cease. At that time the sponge began to distend, and evidently brought on the most violent inflammation, swelling, and acute pain, I recollect to have seen upon any occasion, and which, perhaps, cannot be better described than in the patient's own words ——

" I am extremely sorry (said she) that you

" did not, as you seemed inclined, take off my

" leg above the knee, and cure it in the same

" easy way that you did my other limb; for the

" pain of that operation, and the whole cure,

" was nothing compared with what I now feel

" even in my old ſtump; what muſt I then " endure in the recent one?"

Cold vegeto-mineral water aſſiduouſly applied, and opiates liberally adminiſtered, afforded her ſome relief. A copious ſuppuration ſucceeded, and, by degrees, the granulations ſhot through the fibres of the ſponge until it ſeemed ſo organized as to add greatly to the difficulty of deſtroying, or, by any means, ſeparating it. I regretted much that we had not tried to find ſome intervening ſubſtance which might prevent the bad effects and yet not deſtroy the ſtyptic quality of the ſponge. The muſcular parts, which had been reſerved in the hinder part of the leg, were ſtill found of great uſe, as ſhe had, in the end, a very excellent ſtump.

The appearances of the callus, on examining the amputated limb, were too curious to be omitted. At the end of each fractured bone a callus had begun to form, over every part of which a perioſteum had grown; from this membrane were tendinous and muſcular fibres intermixed, ſo as to unite the ends of the bones. For a ſpace of two inches, which ſhould have been callus, there was no oſſification whatever; but, inſtead of it, only muſcular and and tendinous fibres; nor is it probable there ever would have been even in any habit, much leſs in a woman at her time of life.

The patient frequently, and in the moſt pathetic terms, deſcribed the difference of pain in a ſtump healed by the firſt intention, and the dreſſing of an open ſtump even when nearly healed. In ſhort, nothing but her remarkably ſtrong conſtitution and compoſure of mind could have ſupported her under ſuch tedious ſufferings. For two years ſhe continued to enjoy good health, and then died of an epidemic dyſentery.

As the firſt ſtump was perfectly cured in a month, and the ſecond not in leſs than four months, we have, in this caſe, a ſtrong proof of the ſucceſs of Mr. Alanſon's method, even under very difficult circumſtances, compared with a more open ſtump, although conſiderable delay may be attributed to the ſponge. Every care and attention proved, in this inſtance, inſufficient to prevent ſubſequent hæmorrhage. I recollect alſo three other ſimilar caſes from operating near the affected part; beſides one which required even a ſecond amputation.

All the preceding caſes demonſtrate the pro-

 priety priety of a ſpeedy deciſion whether amputation is or is not neceſſary.

### CASE VII.

Thomas Wilkinſon, a middle-aged man, was become hectical from an old and large ulcer in the leg, with a caries of the bone. The ulcer had ſeveral times bled to an alarming degree, and, from the integuments being thickened and diſeaſed in the vicinity of the ulcer, we judged an amputation below the knee would moſt probably be attended with circumſtances ſimilar to thoſe of Eſther Pearſon's caſe. We likewiſe thought the man too weak to ſuſtain any copious evacuation; and therefore adviſed the operation above the knee, which was done according to Mr. Alanſon's rules, without any ſubſequent hæmorrhage, and the ſtump was healed in a month. This caſe happened ſix years ago, and the patient is now well, and has an uſeful limb.

I have no doubt but this perſon reaped conſiderable benefit from having his leg taken off above, inſtead of below, the knee.

### CASE VIII.

June 11th, 1768, I amputated the leg of John Keighley, aged twenty-five years, above the knee, by a double circular incision, and dressed the stump with lint, digestives, a linen bandage, and woollen cap, as was at that time usual.

On the sixth day the superficial dressings were removed, and, I will venture to say, not without more pain to the patient than is now experienced during the cure. As this was the first limb I amputated, I have no doubt but my utmost efforts were exerted to effect a cure, which was accomplished in two months. This patient died hectical about a year after he left the hospital.

### CASE IX.

About ten or twelve years ago I took off T. Tankard's leg above the knee, according to the directions given by Mr. Gooch in the sixty-fifth volume of the Philosophical Transactions. The stump was sooner healed, and a better one than I had before seen, by the circular incision, and I have reason to believe could bear pressure upon its end; for in one part it is now become come conſiderably thinner, and ſo tender, that the man cannot bear the ſame preſſure that he has been accuſtomed to do with eaſe. He is now under my care again, that I may direct ſome alteration in the artificial leg, or take ſome ſtep to render his ſtump able to bear the uſual preſſure of the machine.

Having found that the bearing of the artificial leg is not at the top of the thigh, I have ordered the addition of a piece of tin to be well padded with a quilted lining, and have added, both before and behind, a broad ſtrap, containing ſeveral ſhort pieces of elaſtic wire. The ſtraps faſten over the ſhoulders, and within, at the bottom of the boot, is an elaſtic cuſhion.

### CASE X.

In March, 1783, I purſued Mr. Alanſon's directions in taking off a boy's leg above the knee. The ſloping inciſion through the muſcles was made with the point of the knife. No retractor was uſed.

By having previouſly marked the dimenſions recited in my former paper, the lips of the wound met with great eaſe: they were drawn together tranſverſely, and united by four ſutures and ſhort ſlips of court plaſter. Three ligatures were left out in ſeparate parts, as ſuited their attachments.

A fine and new flannel circular bandage was applied, which alſo retained two long tow pledgets. The ſtump was placed upon a folded ſheet, and in ſuch poſition as ſeemed eaſieſt to the patient.

When the ſtump was firſt dreſſed, which was on the fifth day, there was very little diſcharge or inflammation; on the tenth day all the ligatures, as well as ſutures, were removed, and the wound very nearly cured. He was out of bed daily; and on the twenty-firſt day the cure was compleated.

I firſt performed Mr Alanſon's operation from verbal directions, before he publiſhed on the ſubject; and from not having attended to the neceſſity of uſing the point of the knife, I cut through a ſmall portion of the integuments—an accident that I have never ſince ſeen.

At that time we united the edges of the wound longitudinally, by which means the ſlit of the teguments, being at the moſt depending part, afforded me a convenient receptacle for all the ligatures, and the ſtump was thereby cloſed throughout its whole ſurface; but being done by by long plafters, inftead of futures, the lips of the wound inflamed more than is now ufual.

In a boy, whofe leg I amputated at the fame place, although the dimenfions prefcribed were not exceeded, the edges of the ftump over-reached, fo as to require a little variation in the retentive means. Only fhort flips of court plafter and tow pledgets, retained by the flannel circular bandage, were ufed, without any futures. Such a contraction took place as prevented any obftacle from the furplus. In another cafe, the difficulty of retraction rendered the lips of the wound not fo eafy of accefs, and in that cafe two long flips of adhefive plafter, as well as the futures, were deemed neceffary, and fully anfwered the purpofe.

### CASE XI.

October 14th, 1785, I took off the leg of Rofe Balmforth, aged twenty-nine years, above the knee, with a flap. She was fo reduced as to make it dubious whether fhe was able to undergo the operation; during which fhe fainted twice. The circumference of the limb exceeded thirteen inches, and was difeafed fo high as not to admit of a flap of fufficient dimenfions.

An

An allowance for the defect was accordingly made by the circular incifion.

Being unable to elevate the flap, I formed it with a fcalpel, though I prefer the catlin.

The cellular membrane was full of a gelatinous fluid, and the mufcles feemed equally difeafed. From the œdema of the whole limb the tourniquet failed to make that effectual preffure to be wifhed, which made it neceffary for the operator to be expeditious; and, upon this occafion, my not ufing a retractor, and fawing through the bone quickly, were ufeful.

The arteries were of neceffity more denuded, by drawing the ligatures, than was defirable, from their cutting through the difeafed parts. I placed the lips of the wound in clofe contact, and they met with fuch facility, that the point of the flap over-reached above half an inch.

I ventured to pafs only two ligatures, and fupported the parts with long tow pledgets, from a want of confidence in a fpeedy union of the wound, and from an idea that the ftate of the parts would require digeftion. An opiate afforded her a good night, and the next morning fhe was much better; but that evening a diarrhœa, to which fhe had been fubject, returned with fuch violence, that her life was defpaired of.

 There

There was alſo a very great diſcharge from the ſtump.

Oct. 17th. She was a little recovered by the uſe of cordial aſtringents and anodyne medicines. The ſtump was dreſſed; and although the diſcharge was copious, and of a ſerous kind, an entire inoſculation had taken place at the ſides where the ſutures were applied, yet at the apex of the flap it was not united, but had contracted, or rather the oppoſite edge was retracted by the flexor muſcles, ſo as to leave an open wound an inch and a half broad. — A long and broad ſlip of adheſive plaſter only prevented any farther retroceſſion. — From what has happened in ſimilar caſes, I have no doubt but an additional ſuture would have been uſeful.

19th. In the evening, the diarrhœa returned with the ſame violence as before, and brought on ſuch fainting and difficulty of breathing, that her death was hourly expected. The diſcharge ſtill continued from the ſtump, and yet the adheſion went on well. Part of the ligatures were caſt off. The adheſive retention was continued.

21ſt. The remaining ligatures and ſutures were removed.

28th.

28th. Although the wound was now reduced, o as to give her little trouble, yet there was ftill no difpofition in it to heal, and fhe remained fo extremely weak, that the other limb was become œdematous, and fwelled to a great fize; nor could fhe bear to fit up long together.

Nov. 12th. Her ftump was perfectly cured; but fhe remained in the houfe for a week or ten days until fhe was able to be taken home.

On inquiry I found fhe recovered her ftrength; but in lefs than two years died confumptive.

This cafe fhows clearly, that although the parts are in a very unfound ftate, and the habit fo reduced as fcarce to make an operation advifeable, the flap operation is fuccefsful, and healing by the firft intention fuffers no material impediment; nay, to this plan I was confident the woman owed her life.

If we could promife that when the edges are allowed to feparate we could afterwards bring them to approximate, I fhould lefs object to fuch a plan; but in general fuch an adhefion of the lips of the wound takes place as to prohibit fuch an attempt.

In one cafe I preferved the flap pofteriorly; but great inconvenience happened from having made it too thick, efpecially as the thigh was

ſo large as to be fifteen inches in circumference. In another caſe I formed a flap at the inſide of the thigh. In this inſtance, the parts where I made the circular inciſion were ſo diſeaſed, that I found it neceſſary to adopt Mr. Gooch's direction to divide the muſcles around the bone, in order to procure a ſufficient retraction of the muſcular parts. Both theſe ſtumps united well, and were cured in a month. In the latter caſe, ten days after the operation, a violent inflammation was brought on the ſtump by putting on a flannel bandage, which, by having been often waſhed, had loſt its elaſticity.

When the flap is made behind, the line of union falls too near the line of the bone, but before the cure is performed that alters.

### CASE XII.

June 16th, 1783, I took off the leg of Abraham Sharpe, of about fifty years of age, above the ancle. The circumference of the limb in that line where the bones were intended to be ſawed through was near ten inches. I marked a flap of about three inches and a half, which, as ſoon as the amputation was performed, was placed in immediate union by four ſutures. A like number of ligatures were placed over the edges of the the ſtump, in ſeparate parts, neareſt their attachment, and the intervening part of the lips cloſed by court plaſter. The limb was placed on its ſide upon a folded ſheet, with the knee a little bended.

The ſtump was not dreſſed until the ſixth day; and the diſcharge was ſo little, that it did not require, at any time, dreſſing more frequently than every other day. At the ſecond dreſſing the ligatures and ſutures were all removed, and the wound was of little conſequence, being no more than what was occaſioned by the ligatures and ſutures, a perfect and firm adheſion of the flap to the ſtump having been formed. The attachment was, however, ſtill preſerved by the application of a long ſlip of ſtrong adheſive plaſter as broad as the flap. I do not know that I ever ſaw a patient ſuffer ſo little; as he never would acknowledge that either during the operation or cure he had had any pain but what was very tolerable. From the day of his admiſſion to that of his being diſcharged cured was exactly three weeks.

This man had for four years had a ſcirrhous tumour in his foot. An ulceration had lately taken place, evidently of a cancerous nature, but the diſeaſe appeared then to be entirely confined fined to below the ancle. Within two years, however, he ſhewed me a large indurated gland on the uppermoſt part of the ſame limb, that increaſed very rapidly, and proved fatal.

In the whole courſe of my practice I do not recollect to have ſeen one inſtance of any long-continued ſucceſs from the removal of a true ulcerated cancer. If in any caſe ſucceſs might be expected, this ſeemed the moſt promiſing. The diſeaſe appeared to be entirely local; the patient's habit of body remarkably ſtrong: he was regular and induſtrious, and reſided in as healthy a ſituation as any in the county.

### CASE XIII.

June 12th, 1788, James Hewit, aged ſeventeen years, had his leg amputated above the ancle with a flap, and the wound was perfectly healed in eighteen days. The limb was meaſured, the tourniquet bolſter applied, and the patient's eyes covered, before he was brought into the operation room.

In forming the flap, the inciſion was made from above downwards. The diviſion of the integuments, muſcles, and all the interoſſeous parts, was made by the ſame catlin. No retractor was uſed, and of courſe the perioſteum was not in any part injured above where the bone was cut through. In ſawing through the bones, I directed the limb to be held low enough for the handle of the inſtrument to be ſufficiently elevated for the ſaw to work obliquely. All the arteries were taken up with the tenaculum. Five ligatures became neceſſary, and as many ſutures, to retain the flap in contact with the ſtump. Court plaſter was uſed as in the laſt caſe; and the parts met with the greateſt facility. Two long tow pledgets and a fine new circular flannel bandage completed the dreſſings. The eaſe of the patient was conſulted by directing his poſition in bed, and the limb was placed on its ſide with the knee bent. Neither opiate nor aperient medicine were indicated.

On the 17th, (the fifth day after the operation), the ſtump was dreſſed, and the diſcharge was greater than uſual, which I attributed to the number of ligatures, as the boy had continued very eaſy; but this diſcharge had by no means impeded the attachment of the flap. For four days after this the ſtump was daily dreſſed, and after that time the diſcharge was ſo much abated as to render a dreſſing every other or every third day ſufficient. In ten days all the ſutures were removed, and all the ligatures, tures, except one, caſt off. The flap continued to be ſupported by a broad ſlip of plaſter, and on the fifteenth day the other ligature was eaſily removed. On the 29th the wound was perfectly cured.

Before the boy was diſcharged I took the dimenſions of his limb. From his knee to the end of the ſtump meaſured eight inches and a half; the breadth of the flap near three inches; and what is rather ſingular, and in favour of marking the length of the flap, both that and the circumference of the limb continued exactly the ſame as before the operation.

Although in this caſe matter might be ſaid to form, yet there was not any ſuch copious diſcharge as either to produce difficulty in the healing of the wound, or to retard its cure. The ſpeedy union of ſuch ſtrong muſcular parts, in my opinion, removes the objection that has been made to ſuch modes of operating. This boy could bear conſiderable preſſure upon his ſtump for ſome time before it was cured; and the end of his ſtump is ſupplied with ſo thick a cuſhion, that it will not very ſoon by preſſure be reduced ſo thin as if no ſuch muſcles had been reſerved.

Mr.

Mr. Douglas, ſurgeon of the forty-fourth regiment, was preſent at this operation, and ſaw the progreſs of the cure; from which, as well as from having ſeen other ſimilar caſes at our Infirmary, he authoriſes me to add his teſtimony and approbation of ſuch a treatment.

### CASE XIV.

In the caſe of Iſaac Hinchliffe, whoſe leg was taken off above the knee, I endeavoured to purſue the intention of Mr. Mynors *; but as ſome variations had occurred to me, I tried them as I ſhall mention. — His dimenſions were marked upon the limb, and a narrow ſlip of card, marked with half inches, was in readineſs to meaſure with during the operation. I preſerved the full quantity of integuments which he directs, by ſetting the ſtrictures at liberty, cloſe to the muſcles, with the point of the amputating knife, which is much more expeditious than diſſecting with the ſcalpel. An equal attention was paid to his direction relative to the quantity of muſcular parts. No retractor was uſed. The edges of the wound met with the greateſt eaſe. A circular bandage of fine Indian callico was applied, and the ſtump was dreſſed, after the poſition of

* Practical Thoughts on Amputations. 12mo.

the patient was accompliſhed, with the tailed bandage, according to Mr. Mynors's rules in every reſpect, except one. It appeared to me that a ſeparation in the edges of the wound muſt take place, unleſs a farther ſupport was added; I therefore applied a long tow pledget over the dreſſings.—Although my directions for the patient to keep upon his ſide were ſtrict, I generally found that he had ſhuffled more upon his back; and when I complained, he replied, that his preſent poſture was eaſy. As his ſtump was not miſplaced, I was leſs anxious to inſiſt upon an alteration.

On the fourth day, at the firſt dreſſing, I found the ſtump much more open than we were accuſtomed to ſee it. The lips of the wound were near two inches aſunder, and ſo firm an adheſion had already taken place, that, in future, little farther approximation could be obtained; hence the patient had not only more pain for many dreſſings, but alſo a broader cicatrix. The only advantage that I could obſerve was, that the ligatures came out a day or two ſooner. I had no doubt but the ſtump would, in all reſpects, have been better, if a greater proportion of muſcular parts had been ſaved, and the edges had been united by ſuture.

The ſtump, in this caſe, did not appear to have an equal defence with thoſe patients on whom we operate after Mr. Alanſon's method; and if the ſtump grows thinner, no diſadvantage can ariſe from forming a thicker cuſhion. Although it is not intended that any principal bearing ſhould be on the end of the ſtump, yet the more we can give it the power of ſupporting this the better; and however it may be ridiculed by ſome, I have no doubt but there are many who have undergone the flap operation below the knee that can bear a great deal of preſſure upon the end of the ſtump. But although I contend that ſuch ability in a ſtump is to be wiſhed for, I by no means recommend the bearing of an artificial leg to be principally upon the end of the ſtump; on the contrary, I think the limb near the affected part has been made to bear more than is neceſſary, and that the more the whole body can be employed in ſupporting and moving this artificial mechaniſm the better.

In an artificial limb below the knee, there ſhould not only be a bearing below, but above the knee*, and that again continued to the top of the

* An artificial leg, for a patient whoſe limb had been taken off near the ancle, was found to receive great advantage from making the bearing not only below and above the knee, but alſo the thigh; and by ſpring ſhoulder ſtraps, the upper parts of the body may be contrived to aſſiſt not only the ſupport, but the motion of the limb.

The connexion between the artificial leg and the ſhoulder ſtraps ſhould be of the ſtrongeſt Ruſſian linen, which may be lined with callico.

For moſt of theſe hints I am indebted to Mr. Mann, a mercer at Bradford, who has been led to take great pains on the ſubject, from having a near relation who required ſuch a machine. He has a moſt excellent mechanical turn, and I much doubt if any perſon has yet made an artificial leg to be compared with that invented by him. I have not ſeen any one except his where the artificial joint of the knee bends in walking, or where the patient can put his artificial leg in the ſtirrup with a bended knee in riding.

Mr. Mann makes it with an exact repreſentation of each joint acting upon natural principles. Mr. Sharpe, and ſome of the moſt eminent ſurgeons in London, have ſeen his contrialſo on the hip; as well as from the addition of ſhoulder ſtraps with ſtrong circular ſtraps. The weight of the leg and ſtraps did not amount to two pounds, or nearly equal thoſe that had been previouſly made on ſuch occaſions.

vance, vance, which is much lighter than the machines of this kind commonly made, and in every respect exceeds those I have examined that have been made in London, Edinburgh, or other places. He warrants it for seven years.

The cases I have selected were chiefly the result of consultation, and were open to the inspection of many strangers as well as pupils, who have universally acknowledged their satisfaction and approbation. Some of the latter have already informed me of the beneficial effects they have experienced from a similar mode of treatment in their own practice.

The more difficult the case is, the more, I think, a preference is due to this method: hence it becomes in a particular manner worthy the attention of military surgeons; and I am persuaded that if the rules and precautions I have laid down are duly observed, the treatment I have recommended will not fail to afford the most ample satisfaction both to the practitioner and the patient.

*Leeds,*

July 13, 1788.

